# A192 'GLYCOCAGED' DRUGS: PHARMACOKINETICS AND TRANSLATIONAL POTENTIAL FOR PEOPLE WITH IBD

**DOI:** 10.1093/jcag/gwad061.192

**Published:** 2024-02-14

**Authors:** W Ma, M Luzentales-Simpson, S C Menzies, C Wang, J Kothandapani, N Haskey, D Gibson, H Brumer, L M Sly

**Affiliations:** The University of British Columbia, Vancouver, BC, Canada; The University of British Columbia, Vancouver, BC, Canada; The University of British Columbia, Vancouver, BC, Canada; The University of British Columbia, Vancouver, BC, Canada; The University of British Columbia, Vancouver, BC, Canada; The University of British Columbia, Vancouver, BC, Canada; The University of British Columbia, Vancouver, BC, Canada; The University of British Columbia, Vancouver, BC, Canada; The University of British Columbia, Vancouver, BC, Canada

## Abstract

**Background:**

Inflammatory bowel disease (IBD) is characterized by chronic inflammation in the gastrointestinal tract. Despite the alarming global increase in IBD burden with 1 in 121 Canadians affected, no cure exists. Systemic administration of anti-inflammatory drugs like corticosteroids is a front-line therapy for inducing remission. However, these drugs are limited in their application by deleterious off-target side effects. To target the drug to sites of inflammation and reduce systemic uptake, we developed a microbiome-cleavable, glycoconjugate drug delivery system, termed GlycoCage. We have demonstrated that GlycoCaged dexamethasone (Dex, a potent steroid), is effective at reducing inflammation at 10-fold lower doses than its uncaged counterpart in the SHIP-deficient (SHIP-/-) mouse model of CD-like intestinal inflammation.

We hypothesize that GlycoCaged technology can expand the therapeutic window of IBD drugs by lowering effective doses and eliminating side effects.

Aims:

1) Quantify Dex in systemic circulation.

2) Assess status of inflammation outside of the gut in SHIP-/- mice treated with caged Dex.

3) Measure decaging activity in people with IBD.

**Methods:**

To compare systemic distribution of Dex to caged Dex, we orally gavaged SHIP+/+ and SHIP-/- mice with 3 mg/kg of Dex or the molar equivalent of caged Dex and collected serum for LC-MS/MS analysis at multiple timepoints post oral gavage. In addition to CD-like ileitis, SHIP-/- mice spontaneously develop enlarged mesenteric lymph nodes (MLNs) and inflamed lungs. After daily treatment of Dex or caged Dex for 2 weeks, MLNs and lungs were collected and analyzed for size and histopathology. We also obtained stool samples from healthy control participants and people with IBD to investigate the prevalence of prodrug-cleaving activity by direct enzymatic activity assay using fluorogenic resorufin as a prodrug proxy. Data from people with IBD were stratified to fecal calprotectin.

**Results:**

Unlike free dexamethasone, caged dexamethasone did not enter systemic circulation or impact inflammation outside of the gut in SHIP-/- mice. All people have decaging activity that is not affected by IBD or the individual’s disease activity.

**Conclusions:**

Our results highlight a novel glycoconjugated prodrug strategy to improve and broaden treatment options for people with IBD. In the future, we will evaluate GlycoCaged Dex in a mouse model of colitis and test the efficacy of other GlycoCaged drugs.

**Funding Agencies:**

CCCGlycoNet

